# Acceptance of sexual minorities, discrimination, social capital and health and well-being: a cross-European study among members of same-sex and opposite-sex couples

**DOI:** 10.1186/s12889-015-2148-9

**Published:** 2015-08-21

**Authors:** Arjan van der Star, Richard Bränström

**Affiliations:** Erasmus University, Rotterdam, The Netherlands; Department of Clinical Neuroscience, Karolinska Institutet, Berzelius väg 3, Floor 6, 171 77 Stockholm, Sweden

## Abstract

**Background:**

Awareness of health disparities based on sexual orientation has increased in the past decades, and many official public health agencies throughout Europe call for programs addressing the specific needs of lesbian, gay and bisexual (LGB) individuals. However, the acceptance of LGB individuals varies significantly in different countries, which potentially influences health and well-being in this population. We explored differences in self-rated health and subjective well-being between individuals living in same-sex and opposite-sex couples. We also examined the effects of discrimination and country-level variations in LGB acceptance on health and well-being and the potential mediating role of social capital in these associations.

**Methods:**

Using the 2010 European Social Survey (*n* = 50,781), 315 individuals living with a same-sex partner were matched and compared with an equal number of individuals living in opposite-sex couples. We performed structural equation modeling analyses to estimate path coefficients, mediations and interactions.

**Results:**

LGB acceptance was significantly related to better self-rated health and subjective well-being among all individuals, and these associations were partially mediated by individual social capital. No differences in these associations were found between individuals living in same-sex and opposite-sex couples. Sexuality-based discrimination had an additional significantly negative effect on self-related health and subjective well-being.

**Conclusions:**

The findings of this study suggest a negative association between exposure to discrimination based on sexual orientation and both health and well-being of individuals living in same-sex couples. Members of same-sex couples and opposite-sex couples alike may benefit from living in societies with a high level of LGB acceptance to promote better health and well-being.

## Background

Eliminating health disparities is a fundamental goal of public health research and practice. Health disparities can be described as differences in the incidence, prevalence, mortality and disease burden between minority and majority population groups [1]. Disparities based on many factors such as age, ethnicity, gender, socioeconomic status, geography and disability have been identified in public health research [1]. In the past several years, public health policy and research have begun to address the substantial health disparities that exist between sexual minority (i.e., lesbian, gay and bisexual; LGB) and heterosexual individuals [2]. In particular, recent studies have revealed large differences in mental health between sexual minorities and heterosexual individuals [3]. A recent review of physical health disparities according to sexual orientation also identified substantial and compelling evidence for physical health problems among LGB individuals compared with heterosexuals [4]. Poor physical and mental health among LGB individuals has been explained by the concept of minority stress. According to Meyer (2007), minority stress among LGB individuals can influence physical and mental health through four main processes: exposure to negative stressful events such as discrimination; the stress of expecting negative events to occur; stress related to the concealment of sexual orientation; and internalization of societal negative attitudes [5]. In his model of minority stress and mental health, Meyer also describes stress-ameliorating factors such as coping and social support, which can reduce the impact of minority stressors.

When looking at the distressing effects of social support and environments in particular, an increasing number of studies have confirmed that structural social environmental discrimination is negatively associated with health among sexual minorities [6–12]. Structural forms of discrimination include unequal marriage legislation, policies extending protections against hate crimes, employment discrimination based on sexual orientation, and the low concentration of same-sex couples. Structural discrimination of LGB individuals varies widely across Europe, as demonstrated in several European-wide surveys [13]. These surveys have shown that in numerous countries LGB people still live in communities where a majority of the population supports discrimination and inequality for sexual minorities. In many countries, LGB people are also subject to legal discrimination concerning basic civil rights, e.g., regarding recognition of same-sex unions. For example, equal marriage rights for sexual minorities have recently been a topic of heated political debate in the United Kingdom and France [14, 15]. In Russia, an “anti-propaganda” law has been adopted to ban public information regarding homosexuality, and many non-governmental organizations have stated that this law violates human rights, i.e., the freedom of expression, assembly and privacy [16]. However, in other countries such as Belgium, the Netherlands, Spain and most Scandinavian countries, equal marriage rights have been legally in place for many years. These significant differences in LGB acceptance and differences in institutional discrimination make cross-European studies particularly suitable for exploring the consequences of structural discrimination, social support and LGB acceptance on health.

Given the minority stress model, discrimination toward sexual minorities on the socio-cultural level, i.e., low LGB acceptance, is likely to have an effect on the intensity of all stressors, as noted by Meyer (2007). Discrimination may lower the ability of LGB individuals to participate in social activities, which leads to increased social exclusion. Furthermore, discrimination may hamper the accessibility of stress-ameliorating social support. In the social sciences, the availability and accessibility of social support have been conceptualized in the theory of social capital. Social capital is commonly used in relation to social inclusion, participation and support. A higher level of social capital, and access to it, has been associated with elevated population health and psychological well-being [17–19]. Two basic elements are often used in most definitions of social capital: a structural component and a cognitive component [17]. The structural component is described as the extent to which societies are formally linked and their members are actively involved in social activities [17]. This element may serve as a bridge for deviations between groups (bridging) or within groups (bonding), leading potentially to social inclusion [17, 20, 21]. The cognitive component captures common societal perceptions and trust between persons within a community based on shared values, attitudes and beliefs [17, 20]. It is common to refer to the cognitive component of social capital as social trust when studying its effects on health. In analyses using structural equation models to describe determinants of health, social trust was found to explain up to 58 % of the total variance in mortality rates across states in the United States [22, 23]. Consistent with the theory of social capital and minority stress, one can hypothesize that low LGB acceptance may lead to the exclusion of LGB individuals from social neighborhood communities and dominant majority groups, lower levels of social trust and support among LGB groups, and lower accessibility of social capital. These processes cumulatively result in greater minority stress and health disparities when LGB individuals are compared with opposite-sex-attracted individuals.

Bonding within the LGB population may serve as a compensation mechanism for the negative impact of social exclusion from majority groups. However, such within-group support is less likely to have as strong a positive effect on LGB individuals as it has on members of ethnic minorities [21]. First, a strong LGB group identity is lacking because of the large degree of diversity within the LGB population [24, 25]. When comparing individuals in similar social contexts larger differences are present between LGB individuals than between LGB and non-LGB persons [24, 25]. Furthermore, the support of strong family ties may be absent for LGB individuals living in low-acceptance settings due to the high risks of abandonment after they disclose their sexual orientation [26].

LGB acceptance may therefore not only affect health directly via lowering discrimination and minority stress but also through the influence of social capital and inclusion and the availability and accessibility of social support. Hence, bonding and bridging social capital may serve as a positive mediator in the hypothesized relation between acceptance and health of sexual minorities. To the best of our knowledge no studies to date have specifically examined the influence of acceptance and social capital on the mental and physical health of LGB individuals.

We explore differences in subjective well-being and self-reported health between sexual minority individuals (members of same-sex couples) and heterosexuals (members of opposite-sex couples) using data from the 2010 European Social Survey (ESS) [27]. We also examined how country-level LGB acceptance, social capital on the individual and country level, and socio-demographic variables affected health and well-being. The specific research questions of the study include: Do levels of health and well-being differ between individuals in same-sex and opposite-sex couples? Is LGB acceptance on the country level associated with health and well-being, and can social capital mediate this association?

## Methods

### Participants and countries

Data were obtained from the fifth ESS on the attitudes, beliefs and behavior patterns of diverse populations from over 30 nations from 2010 based on validated questionnaires [27]. This survey was a biennial multi-country survey that was conducted among 50,781 respondents from 26 European countries; Belgium, Bulgaria, Croatia, Cyprus, Czech Republic, Denmark, Estonia, Finland, France, Germany, Greece, Hungary, Ireland, Lithuania, Netherlands, Norway, Poland, Portugal, Russian Federation, Slovakia, Slovenia, Spain, Sweden, Switzerland, the United Kingdom and Ukraine. The ESS includes variables on a range of social themes such as moral values, security, politics and trust in governments; the results demonstrate significant cross-country variation. The ESS also includes general self-rated health outcomes. In this fifth round, the response rates ranged from 30.5 % (Germany) to 81.4 % (Bulgaria). The survey employs rigorous methodologies. The data were collected by means of hour-long, face-to-face interviews incorporating questions on a variety of core topics repeated from previous rounds of the survey. The survey involved strict random probability sampling of individuals 15 years or older and rigorous translation protocols. Lithuania was excluded from this study due to the combination of a low response rate (39.4 %) and the low number of individuals living in same-sex couples.

### Ethics

In the ESS, national fieldwork organizations are asked to sign and adhere to the International Statistical Institute’s Declaration of Professional Ethics (1985). National and European Union data protection guidelines apply to all data collection methodologies. After the data are collected by national institutes, they are handled anonymously and are openly available on the ESS website after registration. We retrieved anonymous data from the ESS website and used them for this study based on earlier non-study-specific informed consent.

### Propensity-score matching

In total, 329 individuals from same-sex couples and 28,809 individuals from opposite-sex couples were identified in the ESS 2010 dataset. Since the number of individuals living in opposite-sex couples significantly outnumbered the number of individuals living in same-sex couples, we performed propensity-score matching using the R plugin in IBM SPSS Statistics 21 software [28]. This technique reduces bias by accounting for different covariates, which creates more comparable groups. We used the following covariates in the propensity-score matching: age, gender, years of education, country, and if the individual lived in a rural or urban area. The individuals from same-sex couples were matched with the individuals from opposite-sex couples following a most-similar-case approach based on the calculated individual propensity score using the determined covariates. This procedure resulted in a sample of 315 individuals living in same-sex couples and 315 individuals in opposite-sex couples. We examined the standardized mean differences before and after matching to test the level of similarity of the propensity score distributions. The standardized differences between the covariates in the two groups reduced to almost zero: *d* = 0 (Cohen’s *d*). The largest remaining standardized difference was *d* = −0.07 for the age and education variables.

### Measures

In addition to information regarding socio-demographics (i.e., sex, age and years of education), we used a number of items to assess self-rated health, subjective well-being, social capital, LGB acceptance, sexuality-based discrimination, and same-sex partnership. We constructed country scores by using aggregated mean values from all of the ESS 2010 respondents before selecting individuals from same-sex couples and matching them with members of opposite-sex couples.

#### Self-rated health

Perceived health was assessed with the question “How is your health in general?” with five response alternatives (from 0 = “very bad” to 4=”very good”). Single-item assessments of self-rated health of this type have shown by previous studies to be strong predictors of future mortality [29, 30].

#### Subjective well-being

We measured subjective well-being using two variables concerning self-rated happiness and satisfaction with life. Self-rated happiness was measured with the question “Taking all things together, how happy would you say you are?” with an eleven-point response scale (from 0=”extremely unhappy” to 10=”extremely happy”). Self-rated satisfaction was measured with the question “All things considered, how satisfied are you with your life as a whole nowadays?” with an eleven-point response scale (from 0=”extremely dissatisfied” to 10=”extremely satisfied”). The two variables were strongly correlated (*r* = 0.70) and were summed.

#### Social Capital

As described in a literature review of studies related to social capital and health by Islam et al. [31], most authors operationalize social capital as a combination of cognitive (i.e., trust and reciprocity) and structural factors (i.e., informal participation or civic engagement). We assessed the level of social capital using a total of four items. Three questions regarding interpersonal trust were measured on eleven-point scales between two extremes: “Generally speaking, would you say that most people can be trusted (10), or that you can't be too careful in dealing with people (0)?”, “Do you think that most people would try to take advantage of you if they got the chance (0), or would they try to be fair (10)?”, “Would you say that most of the time people try to be helpful (10) or that they are mostly looking out for themselves (0)?” [32, 33]. The fourth item used social participation as a measure of structural social capital [31]: “Compared with other people your age, how often would you say you take part in social activities?” [32, 34, 35]. This question was measured on a five-point response scale with alternatives ranging from 1=”much less than most” to 5=”much more than most”. The responses to all items were standardized and summed to create a score of overall social capital. The internal consistency of the scale was 0.67 (Cronbach’s alpha). We constructed a country-level variable on social capital using the mean country scores of individual social capital.

#### LGB acceptance

We measured the acceptance of LGB individuals using the question “Gay men and lesbians should be free to live their own life as they wish” with a five-point response scale ranging from “Disagree strongly” to “Strongly agree”. We used the mean country-level scores to aggregate the data on LGB acceptance on the country level.

#### Sexuality-based discrimination

Sexuality-based discrimination was dichotomously assessed via the question “Would you describe yourself as being a member of a group that is discriminated against in this country?” When the respondents noted a positive response they were asked to mark the ground(s) on which their group felt discriminated against (e.g., color or race, nationality, religion, language, ethnic group, age, gender, sexuality, disability or other grounds). Data related to sexuality-based discrimination were coded as 0=”No” or 1=”Yes”.

#### Same-sex partnership

Indirect data on sexual orientation were derived from the database by combining data on gender, household composition, gender of household members, and relationships within households. The latter were measured with the question: “Looking at this card, what relationship is he/she to you? (second person in household)”. When respondents answered “Husband/wife/partner” and both the respondent and the indicated second household member had the same gender, they were coded as being in a same-sex couple (i.e., same-sex partnership = 1). Additionally, when the respondent and the second household member had different sexes and were partners, the respondents were coded as being in an opposite-sex couple (i.e., same-sex partnership = 0). If neither of these circumstances were true, sexual preference was coded as “missing”.

### Statistical analysis

**Fig. 1:**
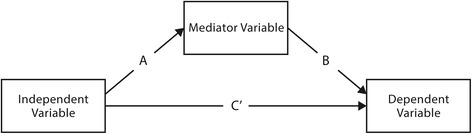
Mediation paths

From the individuals living in same-sex couples (*n* = 315), three cases with missing data in the study variables were excluded from further analysis after matching. Data-centering principles were applied to diminish potential problems with multicollinearity and to avoid contaminating statistical inferences when performing the additional analyses [36]. To test our hypotheses, we used multilevel path analyses with the Mplus 7 software package. These analyses can handle estimating parameters using both individual and country-level data synchronically. We used individual and country-level data to study these variables’ standardized relations to self-rated health and subjective well-being separately. Since a mediating role of individual social capital (mediator) was hypothesized between LGB acceptance (independent variable) and self-rated health and subjective well-being (dependent variables), we added specific mediating paths to the model to test for intermediate processes underlying the directly observed relationships. To this end, we first tested the correlations among the mediator and the dependent and independent variables (α = 0.05). Thereafter, we tested the separate effects of the mediator and independent variable on the dependent variable using a regression equation. Next, we employed the Sobel test to test the mediation model of the mediator and the dependent and independent variables. We accordingly added the mediator to the multilevel path analysis model. In the mediation models (Fig. 1), an *a* path refers to the relation between an independent variable and the mediator, *b* paths were constructed for the relation between the mediator and dependent variables, and *c* paths represent the total association of the independent variable toward the dependent variables. *c’* paths describe the relation between the specific independent variable and the dependent variables after the association has been corrected for the effect of the mediator. We measured the model fit using comparative fit indices (CFI), Tucker and Lewis indices (TLI; non-normed fit index), the root mean square error of approximation (RMSEA), and the standardized root mean square residual (SRMR). We adopted the following recommended levels for the data fit indices as indicators of models: RMSEA ≤0.06, SRMR ≤0.08, and the CFI and TLI ≥0.95 [37, 38].

## Results

Due to matching procedures, the 315 individuals living in same-sex couples were relatively similar to the 315 individuals living in opposite-sex couples for the demographics of age, gender and years of education. Nevertheless, individuals living in same-sex couples reported significantly higher rates of individually perceived social capital compared with individuals in opposite-sex couples. Table 1 presents the respondent characteristics, stratified by sexual orientation. Levels of perceived LGB acceptance and directly experienced discrimination based on sexual orientation were significantly higher in the group of individuals living in same-sex partnerships than in opposite-sex couples.

**Table 1:** Demographics and mean values of main variables, by sexual orientations, after Propensity-Score Matching

	Individuals living in same-sex couples	Individuals living in opposite-sex couples	Absolute mean difference	*P* value
	(*n* = 315)	(*n* = 315)		
*Covariates:*				
Age, years (median; SD)	48.2 (15.0)	49.3 (15.6)	1.1	.40
Gender (male sex %; SD)	60.3 (.49)	60.3 (.49)	.0	1.00
Education, years (mean; SD)	13.4 (4.7)	13.7 (4.9)	.3	.40
*Country level variables:*				
Social capital	20.4 (2.6)	19.4 (2.9)	1.0	<.001
LGB acceptance	3.9 (.5)	3.7 (.5)	.2	<.001
*Individual level variables:*				
Social capital	20.9 (6.2)	19.7 (7.1)	1.2	.03
Discriminated based on sexuality (%)	10.3 (.3)	.0 (.0)	10.3	<.001
Self-rated health	2.9 (.9)	2.9 (.9)	.0	1.00
Subjective well-being	14.4 (3.8)	14.3 (3.8)	.1	.62

Table 2 shows background variables and social capital by country. Country-level LGB acceptance in Europe ranged from 29.6 % in the Russian Federation to 93 % in the Netherlands. In the full sample, mean self-rated health varied from 2.1 in Ukraine to 3.5 in Cyprus. Subjective well-being ranged from 10.9 in Bulgaria to 16.7 in Sweden and Norway. Table 2 shows that directly experienced sexuality-based discrimination was most frequently reported among individuals living in high LGB acceptance countries. Discriminated individuals were younger and had longer education, see Table 3. The individuals living in same-sex couples reported higher levels of sexuality-based discrimination than individuals living in opposite-sex couples.

**Table 2:** Background variables, country-level acceptance of sexual minorities, and social capital by country

	Sample characteristics								Country characteristics	
Country	N	Gender	Age, years	Education, years	Sexuality-based discrimination	Social Capital (Individual)	Self-rated	Subjective well-being	LGB Acceptance	Social Capital (Country mean)
		(% male)	(mean; SD)	(mean; SD)	(%)	(mean; SD)	(mean; SD)	(mean; SD)	(%)	
Belgium	32	37.5	34.6 (15.9)	13.4 (3.8)	9.4	20.6 (5.8)	3.2 (.68)	15.7 (2.0)	87.2	20.5
Bulgaria	18	38.9	42.4 (13.7)	12.0 (4.2)	.0	16.3 (5.6)	2.4 (1.2)	10.9 (5.3)	53.9	16.1
Croatia	12	33.3	34.8 (17.1)	12.3 (4.7)	.0	23.1 (9.5)	3.0 (1.3)	13.8 (5.9)	39.6	17.3
Cyprus	21	47.6	29.9 (16.8)	12.6 (8.8)	.0	15.5 (6.5)	3.5 (.75)	15.0 (3.4)	58.5	16.0
Czech Republic	21	28.6	26.1 (10.1)	13.0 (2.3)	.0	17.6 (6.8)	3.1 (.89)	13.7 (2.9)	65.6	17.9
Denmark	38	15.8	33.2 (12.8)	16.6 (7.3)	7.9	27.0 (4.0)	3.3 (.84)	17.2 (1.9)	90.1	25.0
Estonia	16	50.0	33.7 (16.5)	13.4 (2.2)	.0	19.2 (5.7)	2.3 (.78)	11.6 (4.7)	42.9	20.8
Finland	24	41.7	33.9 (12.5)	15.3 (4.7)	16.7	24.6 (5.3)	2.9 (.90)	15.9 (2.7)	74.7	23.5
France	14	35.7	31.8 (13.6)	14.4 (4.2)	.0	21.2 (4.3)	2.5 (.86)	13.6 (3.1)	82.4	19.6
Germany	56	37.5	37.4 (15.7)	13.9 (4.4)	3.6	20.5 (4.5)	2.7 (.90)	15.0 (3.6)	82.3	19.9
Greece	29	51.7	32.8 (18.5)	10.8 (3.4)	.0	14.3 (6.6)	3.0 (.78)	11.0 (4.3)	52.5	15.3
Hungary	12	41.7	33.1 (11.6)	14.8 (4.1)	.0	16.6 (9.3)	2.3 (1.2)	11.8 (5.0)	48.3	17.6
Ireland	93	51.6	32.4 (15.3)	14.3 (3.5)	1.1	21.2 (5.5)	3.2 (.90)	13.6 (4.1)	52.4	20.8
Netherlands	28	42.9	28.4 (13.6)	15.0 (4.6)	25.0	22.0 (5.2)	2.7 (.76)	16.1 (1.7)	93.0	22.7
Norway	19	31.6	28.3 (15.3)	14.8 (3.0)	10.5	25.4 (4.6)	3.3 (.67)	16.7 (2.4)	83.4	24.7
Poland	15	26.7	34.5 (16.0)	13.3 (4.0)	.0	19.8 (7.4)	2.7 (.96)	15.7 (3.7)	48.8	17.3
Portugal	16	68.8	40.1 (15.9)	9.4 (5.8)	.0	18.2 (4.2)	2.4 (.72)	12.4 (2.9)	64.8	16.7
Russian Federation	18	27.8	34.0 (15.5)	13.5 (4.1)	.0	18.5 (8.1)	2.4 (.80)	12.8 (3.5)	29.6	17.6
Slovakia	15	53.3	35.3 (15.0)	13.7 (3.6)	.0	14.0 (6.3)	2.7 (.72)	12.9 (3.2)	42.0	16.3
Slovenia	13	38.5	35.5 (15.7)	11.4 (2.0)	.0	14.2 (8.6)	2.3 (.63)	13.1 (3.8)	52.6	16.9
Spain	19	31.6	31.1 (16.4)	14.8 (7.6)	15.8	16.6 (6.1)	2.4 (1.0)	13.9 (2.8)	81.4	19.4
Sweden	17	35.3	41.6 (16.1)	14.7 (4.9)	5.9	22.6 (5.3)	3.1 (.70)	16.7 (2.4)	90.2	23.9
Switzerland	18	38.9	27.1 (13.0)	12.9 (3.5)	5.6	21.5 (5.9)	3.1 (.73)	15.7 (2.5)	82.6	22.0
Ukraine	23	34.8	38.2 (15.4)	10.3 (3.9)	.0	20.0 (5.8)	2.1 (.61)	12.7 (3.5)	30.9	17.4
United Kingdom	30	33.3	33.4 (16.1)	13.7 (4.1)	16.7	21.3 (6.8)	3.2 (1.2)	15.5 (3.1)	85.0	21.1

**Table 3:** Bivariate correlations between variables at individual level

		1.	2.	3.	4.	5.	6.	7.
1. Age	Spearman’s rho coefficient	−						
2. Gender^a^	Spearman’s rho coefficient	−0.094^*^	−					
3. Educational years	Spearman’s rho coefficient	−0.252^**^	−0.069	−				
4. Same-sex partnership^b^	Spearman’s rho coefficient	−0.022	0.001	−0.016	−			
5. Discrimination^c^	Spearman’s rho coefficient	−0.139^**^	0.019	0.172^**^	0.231^**^	−		
6. Individual social capital	Spearman’s rho coefficient	0.013	−0.173^**^	0.281^**^	0.127^*^	0.074	−	
7. Self-rated health	Spearman’s rho coefficient	−0.353^**^	−0.035	0.240^**^	0.011	−0.020	0.204^**^	−
8. Subjective well-being	Spearman’s rho coefficient	−0.087^*^	−0.054	0.155^**^	0.045	0.052	0.450^**^	0.332^**^

^a^1 = male, 0 = female;

^b^1 = Individual living with same-sex partner, 0 = Individual living with opposite-sex partner;

^c^1 = Discriminated against based on sexuality, 0 = Not discriminated against based on sexuality

*Correlation is significant at the 0.05 level (2-tailed);

**Correlation is significant at the 0.01 level (2-tailed);

Additionally, being in a same-sex partnership was positively linked to individual social capital. Individual social capital itself was positively associated with years of education, self-rated health, and subjective well-being. Younger and individuals with longer education reported better self-rated health and subjective well-being.

**Fig. 2:**
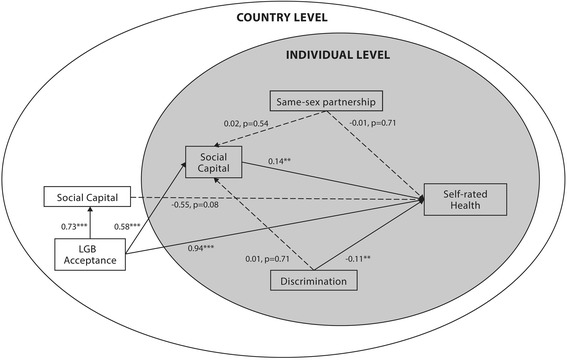
Multilevel structural equation model with self-rated health as dependent variable. Solid lines represent significant relationships with standardized β coefficients; Interrupted lines represent non-significant relationships with standardized β coefficients and *p* values. *P value is smaller than or equal to 0.05; **P value is smaller than or equal to 0.01; ***P value is smaller than or equal to 0.001

**Fig. 3:**
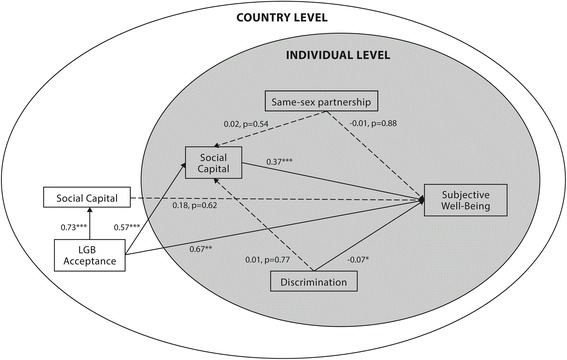
Multilevel structural equation model with subjective well-being as dependent variable. Solid lines represent significant relationships with standardized β coefficients; Interrupted lines represent non-significant relationships with standardized β coefficients and *p* values. *P value is smaller than or equal to 0.05; **P value is smaller than or equal to 0.01; ***P value is smaller than or equal to 0.001

Table 4 shows results from the multilevel structural equation models in regard to outcomes on self-rated health and subjective well-being. For self-rated health, the model’s goodness of fit was found to be sufficient when looking at RMSEA (0.188), TLI (−0.979) and SRMR (0.006) for self-rated health (Fig. 2). However, the comparative fit index was rather small (0.884) and the model predicted 18 % of all variance (R^2^ = 0.18; standard error (SE) = 0.04; *p* < 0.001). The model for subjective well-being, shown in Fig. 3, was sufficient when looking at the goodness of fit scores – RMSEA (0.139) and SRMR (0.007) – but was less suitable when looking at the non-normed fit index and the comparative fit index (−0.214 and 0.929, respectively). This model predicted 15 % of all data variance in subjective well-being (R^2^ = 0.15; SE = 0.03; *p* < 0.001) for the sample’s respondents.

**Table 4:** Standardized beta values for the multivariate models

	Model I				Model II		
	Self-Rated Health				Subjective Well-Being		
	Path	Standardized Beta	SE	*P* value	Standardized Beta	SE	*P* value
Independent variables:		Dependent variables:					
Within level	Self-Rated Health				Subjective Well-Being		
Individual Social Capital	b	0.136	0.045	0.003	0.366	0.041	0.000
Education	c’	0.147	0.041	0.000	0.043	0.045	0.341
Age	c’	−0.340	0.041	0.000	−0.094	0.053	0.078
Gender	c’	−0.063	0.031	0.045	0.001	0.035	0.982
Same-sex partnership	c’	−0.016	0.044	0.710	−0.006	0.042	0.880
Discrimination	c’	−0.112	0.033	0.001	−0.070	0.031	0.024
	Social Capital at individual level				Social Capital at individual level		
Education	a	0.171	0.037	0.000	0.174	0.037	0.000
Age	a	0.052	0.041	0.204	0.047	0.041	0.250
Gender	a	−0.057	0.038	0.133	−0.062	0.039	0.110
Same-sex partnership	a	0.022	0.036	0.536	0.023	0.037	0.540
Discrimination	a	0.009	0.025	0.708	0.007	0.025	0.769
Between level							
	Self-Rated Health				Subjective Well-Being		
LGB acceptance	c'	0.938	0.205	0.001	0.668	0.247	0.007
Country-level Social Capital	b	−0.552	0.316	0.081	0.177	0.359	0.623
	Social Capital at individual level				Social Capital at individual level		
LGB acceptance	a	0.579	0.160	0.000	0.574	0.161	0.000
	Social Capital at individual level				Social Capital at individual level		
LGB acceptance	a	0.732	0.081	0.000	0.732	0.081	0.000

Being in a same-sex partnership did not significantly contribute to the prediction of self-rated health and subjective well-being, after accounting for all covariates. However, higher levels of individual social capital predicted both better self-rated health and subjective well-being (see the β values in Table 4). Nevertheless, we recovered different results for country-level social capital. The between-level (comparing countries as clusters) path coefficients of social capital on the country level for the prediction of self-rated health and subjective well-being were not significant.

In the structural equation models, individual social capital is not significantly mediating the relation of same-sex partnership with self-rated health and subjective well-being. To further assess the moderating effect of living in a same-sex partnership on the models’ various *c’* paths, we added interaction terms between same-sex partnerships and other variables to the models. None of the estimates for these interaction terms turned out to be statistically significant and were excluded from the final models.

After accounting for all covariates, LGB acceptance contributed positively to self-rated health and subjective well-being. In these models, LGB acceptance was found to be the strongest predictor of subjective well-being; it also predicted self-rated health, even when mediation through social capital was added to the model. Moreover, the structural equation models revealed that individual social capital was a significant mediator in the relation of LGB acceptance to self-rated health and subjective well-being. However, we did not observe any mediating effects of social capital in the relation of the demographical variables on self-rated health and subjective well-being. Nevertheless, a significant mediating effect was found for individual social capital when linking education with self-rated health and subjective well-being.

As Table 4 shows, country-level social capital was also significantly positively associated with LGB acceptance. On the other hand, for country-level social capital, no significant relations are found with any of the dependent variables.

When further exploring the role of discrimination, we found a significant estimated path coefficient of sexuality-based discrimination to self-rated health and, to a lesser extent, subjective well-being. The addition of interaction terms with the discrimination variable only revealed a significant interaction with country-level social capital in their relations with self-rated health.

## Discussion

Social capital on an individual level was found to be positively related to subjective well-being and self-rated health among individuals living as couples. No significant difference was found between individuals in same-sex or opposite-sex couples in terms of subjective well-being and self-reported health or in associations between social capital and these outcomes. The absence of differences in social capital between individuals living in same-sex or opposite-sex couples may be due to the good integration of LGB individuals in their social contexts, which is consistent with previous research findings [24, 25]. As addressed in the introduction section, bonding within the LGB population may serve as a compensation mechanism for the negative impact of social exclusion from majority groups. The results suggest that within-group support may play a greater role than hypothesized in this compensation effect for individuals in same-sex couples in particular.

Furthermore, direct exposure to sexuality-based discrimination was inversely linked to both self-rated health and subjective well-being. This effect of directly experienced discrimination on health and well-being supports the theory of minority stress as a predictor of health disparities among LGB individuals, as previously noted in the literature [5]. A potential underlying mechanism could be that sexuality-based discrimination might mainly occur just outside the social (neighborhood) circles of sexual minority individuals; these individuals, and especially those living in couples, may be well supported, interconnected and accepted within their direct non-LGB social environments. Studies of multilevel social capital with aggregated data that do not separately or contextually measure their data may not capture such mechanisms [17, 20].

We also found that country-level LGB acceptance was positively and strongly associated with both subjective well-being and self-rated health among all individuals living in couples. This finding held true both directly and indirectly through individual social capital as a mediator. Social capital on the country level was not identified as a mediator in the association between LGB acceptance and the level of health and well-being experienced by an individual. For members of couples across Europe, LGB acceptance may have other primary direct and indirect links to health and well-being distinct from social capital.

Additionally, individual social capital was found to mediate the relation between education and self-rated health. However, we did not find any significant relation between education and subjective well-being. Gender, age, sexuality-based discrimination and same-sex partnership were not significantly associated with the level of individual social capital. Therefore, no mediating effect of individual social capital was observed for these factors in their association with subjective well-being and self-rated health. Although the results did not take into account income level, the link between education and individual social capital suggests that social capital on the individual level may be an important mechanism underlying socioeconomic inequalities in health, which is consistent with the sociological literature [39, 40].

Acceptance appeared to mainly have a direct positive effect on the health and well-being of couple members, and it varied significantly between European countries. Sexuality-based discrimination negatively affected health and well-being and was not alleviated by social capital. This finding may indicate that the impact of discrimination, social exclusion, mistrust, minority stress and a lack of social support might not be enough to explain the processes of how LGB acceptance affects health and well-being.

### Limitations

We did not gather any data on individuals living without a partner or on the self-reported sexual and gender identities of individuals. The ESS is mainly focused on collecting social and political data and does not include any specific questions regarding sexual identity. As a result, we did not identify self-identified homosexual or bisexual individuals. Since membership in a couple might provide considerable protection from the impact of discrimination and other stressors, which are not available to single people, our results are not representative of the wider LGB group. Furthermore, individuals from same-sex couples plausibly have overcome many of the stressful issues related to self-acceptance of their sexual identity and its disclosure to others, which are often associated with a range of mental problems (e.g., fear of threat and violence, stigma and abandonment by family and friends) [26]. The literature describes that identity self-acceptance and disclosure to others both have beneficial effects on mental health [26]. The average state of disclosure of sexual orientation by the selected individuals from same-sex couples is likely to be higher than the average in an overall sample of self-identified LGB individuals and therefore could (partially) explain the lack of differences between gay/lesbian and heterosexual individuals found in our study. Furthermore, our selection methods do not reflect the variety in the LGB community [24, 25] and did not capture the significant variance in the mental health of all subgroups [41].

Health inequalities have been addressed based on various factors in research such as age, ethnicity, gender and socioeconomic status. However, no useful data on income were available for the entire sample. As a result, the findings were not corrected for either income level or other indicators of prosperity. Since education has a significant relationship with individual social capital, subjective well-being and self-rated health, income is likely to have a significant impact as well. Furthermore, prosperity (and/or religion) could potentially be correlated with LGB acceptance on the country level. Furthermore, no data were available on other potential confounding factors such as geographical, financial and social accessibility to health care services.

Although single measures of self-rated health were strong predictors of mortality [29, 30], a specific stress-related scale could have been more sensitive at capturing subjective well-being in this study.

In the study, data on social capital were aggregated at the country level. Social capital, however, is a concept that expresses itself on the neighborhood level [17]. Although the questions in the ESS that were used to construct the social capital variables did not incorporate a particular frame of reference, it is likely that respondents used their direct social environments as a frame of reference when answering these questions. Furthermore, the ESS did not include separate questions to measure the more contextual aspects of social capital instead of using aggregated individual data for the clustered (country) level [20]. For LGB individuals, this integration with their neighborhood communities instead of their LGB peers may be distinct from that of heterosexual individuals. Plausibly, therefore, our results reveal no significant associations between country-level social capital and the dependent variables despite the fact that LGB acceptance was significantly linked to both country-level social capital and the dependent variables.

Additionally, for some countries (e.g., the Czech Republic and Hungary) very few individuals living in same-sex couples participated. This fact limits our ability to generalize our findings regarding the association between LGB acceptance on health and well-being of sexual minorities across all European countries.

## Conclusions

Our findings provide additional arguments for policy makers across Europe to support the promotion of LGB acceptance in order to promote physical and mental health for couples living together, partially through the bonding effects of social capital. The results of the study call for additional measures to equalize the pronounced differences in LGB acceptance across European countries and improve tolerance and human rights situation in all countries.

Since participation in social networks of all kinds has been in sharp decline for decades [42], indirect effects of LGB acceptance through social capital could diminish in the coming years. However, the direct effects of LGB acceptance on subjective well-being and self-rated health would remain. This research provides evidence that increased LGB acceptance can stimulate bonding within societies and help to diminish differences and promote diversity [18]. Public health interventions should focus on stimulating solidaristic individualism as a contemporary phenomenon, which is characterized by individuals’ willingness to trust and support those different from them [18]. Additional sociological research involving social LGB acceptance, health and well-being should focus on the particular characteristics of sexuality-based discrimination and the association between sexuality-based discrimination and LGB acceptance. Additionally, the use of self-reported data on sexual orientation and the aggregation of contextual social capital data at lower community levels is expected to more precisely shed light on the social effects of LGB acceptance on health.

## References

[1] Whitfield KE, Bogart LM, Revenson TA, France CR (2013). Introduction to the second special section on health disparities. Ann Behav Med.

[2] US Institute of Medicine (2011). The Health of Lesbian, Gay, Bisexual, and Transgender People: Building a Foundation for Better Understanding.

[3] Meyer I (2003). Prejudice, social stress, and mental health in lesbian, gay, and bisexual populations: conceptual issues and research evidence. Psychol Bull.

[4] Lick DJ, Durso LE, Johnson KL (2013). Minority stress and physical health among sexual minorities. Perspect Psychol.

[5] Meyer I, Meyer I (2007). Prejudice and Discrimination as Social Stressors. The Health of Sexual Minorities.

[6] Hatzenbuehler ML (2011). The social environment and suicide attempts in lesbian, gay, and bisexual youth. Pediatrics.

[7] Hatzenbuehler ML, Bellatorre A, Lee Y, Finch BK, Muennig P, Fiscella K (2014). Structural stigma and all-cause mortality in sexual minority populations. Soc Sci Med.

[8] Hatzenbuehler ML, Corbin WR, Fromme K (2011). Discrimination and alcohol-related problems among college students: a prospective examination of mediating effects. Drug Alcohol Depend.

[9] Hatzenbuehler ML, Jun HJ, Corliss HL, Austin SB (2014). Structural stigma and cigarette smoking in a prospective cohort study of sexual minority and heterosexual youth. Ann Behav Med.

[10] Hatzenbuehler ML, Keyes KM, McLaughlin KA (2011). The protective effects of social/contextual factors on psychiatric morbidity in LGB populations. Int J Epidemiol.

[11] Hatzenbuehler ML, McLaughlin KA, Keyes KM, Hasin DS (2010). The impact of institutional discrimination on psychiatric disorders in lesbian, gay, and bisexual populations: a prospective study. Am J Public Health.

[12] Pachankis JE, Hatzenbuehler ML, Starks TJ (2014). The influence of structural stigma and rejection sensitivity on young sexual minority men’s daily tobacco and alcohol use. Soc Sci Med.

[13] Bränström R, van der Star A (2013). All inclusive Public Health – what about LGBT populations?. Eur J Public Health.

[14] Lichfield J. France: Huge gay marriage protest turns violent in Paris. In: The Independent, 2013*.* Retrieved on June 6, 2013, from http://www.independent.co.uk/news/world/europe/france-huge-gay-marriage-protest-turns-violent-in-paris-8632878.html#

[15] Osborn A: British PM’s party split as first gay marriage vote passes. In: Reuters, 2013. Retrieved on June 6, 2013, from http://www.reuters.com/article/2013/02/05/us-britain-cameron-idUSBRE91400820130205

[16] International A (2013). Freedom under threat Clampdown on Freedoms of ExpressIon.

[17] Subramanian SV, Kim DJ, Kawachi I (2002). Social trust and self-rated health in US communities: a multilevel analysis. J Urban Health.

[18] Wakefield SE, Poland B (2005). Family, friend or foe? Critical reflections on the relevance and role of social capital in health promotion and community development. Soc Sci Med.

[19] Rios R, Aiken LS, Zautra AJ (2012). Neighborhood Contexts and the Mediating Role of Neighborhood Social Cohesion on Health and Psychological Distress Among Hispanic and Non-Hispanic Residents. Ann Behav Med.

[20] McKenzie K, Whitley R, Weich S (2002). Social Capital and mental health. Br J Psychiatry.

[21] Kawachi I, Takao S, Subramanian SV (2013). Global Perspectives on Social Capital and Health.

[22] Kawachi I, Berkman LF, Berkman LF, Kwachi I (2000). Social cohesion, social capital, and health. Social Epidemiology.

[23] Kawachi I, Kennedy BP, Lochner K, Prothrow-Stith D (1997). Social capital, income inequality, and mortality. Am J Public Health.

[24] Diaz RM, Ayala G, Bein E, Henne J, Marin BV (2001). The impact of homophobia, poverty, and racism on the mental health of gay and bisexual Latino men: findings from 3 US cities. Am J Public Health.

[25] Herek GM, McLemore KA (2012). Sexual Prejudice. Annu Rev Psychol.

[26] Corrigan P, Matthews A (2003). Stigma and disclosure: Implications for coming out of the closet. J Ment Health.

[27] European Social Survey: ESS Round 5 Data. Oslo: Norway Norwegian Social Science Data Services Data Archive; 2010. Retrieved on January 29, 2013 at http://www.europeansocialsurvey.org/data/download.html?r=5

[28] Thoemmes F. Propensity score matching in SPSS. ArXiv preprint arXiv. 2012;1201(6385).

[29] DeSalvo KB, Bloser N, Reynolds K, He J, Muntner P (2006). Mortality Prediction with a Single General Self‐Rated Health Question. J Gen Intern Med.

[30] Idler EL, Benyamini Y (1997). Self-rated health and mortality: a review of twenty-seven community studies. J Health Soc Behav.

[31] Islam MK, Merlo J, Kawachi I, Lindström M, Gerdtham UG (2006). Social capital and health: Does egalitarianism matter? A literature review. Int J Equity Health.

[32] Halman L, Luijkx R (2006). Social capital in contemporary Europe: evidence from the European Social Survey. Port J Soc Sci.

[33] Reeskens T, Hooghe M (2008). Cross-cultural measurement equivalence of generalized trust. Evidence from the European Social Survey (2002 and 2004). SOCI.

[34] Cramm JM, van Dijk H, Lötters F, van Exel J, Nieboer AP (2011). Evaluating an integrated neighbourhood approach to improve well-being of frail elderly in a Dutch community: a study protocol. BMC Res Notes.

[35] Guillen L, Coromina L, Saris WE (2011). Measurement of social participation and its place in social capital theory. SOCI.

[36] Kraemer HC, Blasey CM (2004). Centring in regression analyses: a strategy to prevent errors in statistical inference. Int J Methods Psychiatr.

[37] Iacobucci D (2010). Structural equations modeling: Fit indices, sample size, and advanced topics. J Consum Psychol.

[38] Bagozzi RP, Yi Y (2012). Specification, evaluation, and interpretation of structural equation models. J Acad Market Sci.

[39] Lomas J (1998). Social capital and health: implications for public health and epidemiology. Soc Sci Med.

[40] Grundy E, Sloggett A (2003). Health inequalities in the older population: the role of personal capital, social resources and socio-economic circumstances. Soc Sci Med.

[41] Lyons A, Hosking W (2014). Health Disparities Among Common Subcultural Identities of Young Gay Men: Physical, Mental, and Sexual Health. Arch Sex Behav.

[42] Putnam RD (1995). Bowling alone: America’s declining social capital. J Democr.

